# Number of Sentinel Medical Institutions Needed for Estimating Prefectural Incidence in Influenza Surveillance in Japan

**DOI:** 10.2188/jea.JE20130077

**Published:** 2014-05-05

**Authors:** Shuji Hashimoto, Miyuki Kawado, Yoshitaka Murakami, Akiko Ohta, Mika Shigematsu, Yuki Tada, Kiyosu Taniguchi, Masaki Nagai

**Affiliations:** 1 Department of Hygiene, Fujita Health University School of Medicine, Toyoake, Aichi, Japan; 1 藤田保健衛生大学医学部衛生学講座; 2 Department of Medical Statistics, Shiga University of Medical Science, Otsu, Japan; 2 滋賀医科大学医学部医療統計学部門; 3 Department of Public Health, Saitama Medical University Faculty of Medicine, Moroyama, Saitama, Japan; 3 埼玉医科大学医学部公衆衛生学講座; 4 Infectious Disease Surveillance Center, National Institute of Infectious Diseases, Tokyo, Japan; 4 国立感染症研究所感染症疫学センター; 5 Department of Clinical Research, National Mie Hospital, Tsu, Japan; 5 国立病院機構三重病院臨床研究部

**Keywords:** surveillance, infectious disease, influenza, epidemiology

## Abstract

**Background:**

The sentinel surveillance system in Japan provides estimates of nationwide influenza incidence. Although prefectural influenza incidences can be estimated using data from the current surveillance system, such estimates may be imprecise.

**Methods:**

We calculated the numbers of sentinel medical institutions (SMIs) needed in the surveillance system to estimate influenza incidences in prefectures, under the assumption that the standard error rates in 75% of influenza epidemic cases are less than 10%. Epidemic cases observed in 47 prefectures during the 2007/2008, 2008/2009, and 2009/2010 seasons, respectively, were used.

**Results:**

The present total number of SMIs was 6669. With respect to current standards, the increases required in prefectures ranged from 0 to 59, and the total increase required in the number of SMIs was 1668.

**Conclusions:**

We used sentinel surveillance data for Japan to calculate the number of SMIs required to estimate influenza incidence in each prefecture.

## Abstract (ja)

**【背景】:**

インフルエンザの全国罹患数推計値は感染症発生動向調査システムから得られる。現行システムのデータから都道府県別罹患数が推計できるが、その精度は十分でない。

**【方法】:**

感染症発生動向調査における都道府県別インフルエンザ罹患数推計に必要な定点医療施設数を算定した。その算定にあたって、インフルエンザ流行ケースの75％において、インフルエンザ罹患数推計値の標準誤差率が10％未満となることを仮定した。インフルエンザ流行ケースとして、3シーズン（2007/2008年、2008/2009年、2009/2010年）の47都道府県で観察されたものを用いた。

**【結果】:**

定点医療施設の標準数は6,669であった。都道府県別インフルエンザ罹患数推計に必要な定点医療施設数として、この標準数からの増加は各都道府県で0～59施設、合計で1,668施設であった。

**【結論】:**

感染症発生動向調査データに基づいて、各都道府県におけるインフルエンザ罹患数推計に必要な定点医療施設数を求めた。

## INTRODUCTION

Many countries have created systems for sentinel surveillance of infectious diseases.^1^^–^^5^ Such systems provide information that is essential for minimizing the burden and impact of an influenza outbreak, but incidence is not obtained directly using data from sentinels.^4^^,^^6^ In Japan, sentinel surveillance of influenza is done as part of the National Epidemiological Surveillance of Infectious Diseases (NESID).^7^ Research has determined the number of sentinel medical institutions (SMIs) required to estimate nationwide influenza incidences using sentinel surveillance data.^8^ Moreover, NESID guidelines specify the method for selecting SMIs.^9^ Prefectural governments select SMIs (about 3000 in pediatrics and about 2000 in internal medicine) according to the guidelines. Nationwide influenza incidence is estimated using data from SMIs.^10^^–^^12^

Countermeasures against influenza epidemics are planned and implemented locally and nationwide.^13^ Incidence estimates for local areas would be useful if their accuracy was above a certain threshold. Our goal was to use data from sentinel surveillance to obtain accurate estimates of influenza incidence in each prefecture of Japan. Although influenza incidence can be estimated in each prefecture on the basis of the current surveillance system, such estimates may be imprecise.^6^ In previous studies of Japan, the precision of nationwide influenza incidence estimates was examined and discussed, but there have been no such studies at the prefecture level.^6^^,^^8^^,^^10^^,^^11^

In the present study, we used sentinel surveillance data from Japan to determine the number of SMIs required for estimating influenza incidence in each prefecture.

## METHODS

### Influenza surveillance in Japan

The NESID in Japan has been described elsewhere.^5^^–^^7^ It is organized by the Ministry of Health, Labour and Welfare (MHLW) and encompasses the sentinel surveillance system for influenza. Prefectural governments select SMIs for the influenza surveillance system, and each SMI reports the weekly numbers of influenza cases to the area health center. Health centers notify prefectural governments and the MHLW by using an online computer network.

### Surveillance data and method for estimating incidence

After obtaining permission from the National Institute of Infectious Diseases of Japan, we used SMI reports of influenza data at the NESID for 3 seasons: 2007/2008 (from week 36 of 2007 to week 35 of 2008), 2008/2009 (from week 36 of 2008 to week 27 of 2009), and 2009/2010 (from week 28 of 2009 to week 12 of 2010). There were epidemics of A(H1N1)pdm09 in the 2009/2010 season.^14^ The numbers of medical institutions were obtained from the National Survey of Medical Care Institutions conducted by the MHLW in 2008.^15^

The method for estimating influenza incidence used in the NESID surveillance system was previously described.^6^^,^^11^ Influenza incidence in each prefecture, by type of medical institution, was estimated as the total number of influenza patients in SMIs divided by the proportion of SMIs to all medical institutions. Incidence in each prefecture was estimated as the total of influenza incidence estimates for all types of medical institution. Medical institutions were divided into 4 types, as follows: (1) hospital pediatrics departments, (2) pediatrics clinics, (3) internal medicine clinics with a secondary pediatrics practice, and (4) hospital departments of internal medicine and internal medicine clinics with no pediatric practice. These are classified as the first, second, third, and fourth types of medical institution, respectively. The Appendix shows the method of estimating incidence in detail.

### Method of determining the standard number of SMIs

The Japan influenza surveillance system includes 2 types of SMIs.^7^^,^^9^ The first comprises hospital pediatrics departments and pediatrics clinics (pediatrics SMI), ie, the first and second types of medical institution. The second comprises internal medicine clinics with a secondary pediatrics practice, hospital departments of internal medicine, and internal medicine clinics with no pediatrics practice (internal medicine SMI), ie, the third and fourth types of medical institution. We calculated the standard number of pediatrics and internal medicine SMIs in prefectures according to the method specified in the NESID guideline. As shown in Table 1, the standard numbers of pediatrics and internal medicine SMIs in areas covered by health centers in prefectures were determined in relation to population size.^7^^,^^9^ We divided the standard numbers of pediatrics SMIs into the first and second types of medical institution, proportional to the numbers of all medical institutions. Also, the standard numbers of internal medicine SMIs were divided into the third and fourth types of medical institution.

**Table 1.** Standard numbers of SMIs in areas covered by health centers, by population size

	Area covered by health center	

	Population size	Standard number of SMIs
Pediatrics SMIs	<30 000	1
	30 000–74 999	2
	≥75 000	3 + (π − 75 000)/50 000

Internal medicine SMIs	<75 000	1
	75 000–124 999	2
	≥125 000	3 + (π − 125 000)/100 000

SMI: sentinel medical institution.

π is population size.

### Method for determining the number of SMIs needed for estimating prefectural influenza incidence

As a condition for precise estimation of incidences in prefectures, we assumed that standard error rates for critical proportions of influenza epidemic cases were less than 10%. The standard error rate is defined as the standard error divided by the incidence estimate and was used as an index of the precision of incidence estimates in a previous report that calculated the numbers of SMIs needed for estimating nationwide influenza incidence.^8^ The critical proportions of influenza epidemic cases were given as 25%, 50%, 75%, and 90%. We assume a critical proportion of 75% in our proposal. Influenza epidemic cases were represented as means and standard deviations (SDs) of the numbers of influenza patients at all medical institutions, which were obtained using data from SMI reports in 47 prefectures for the 2007/2008, 2008/2009, and 2009/2010 seasons. Out of 141 epidemic cases (47 prefectures multiplied by 3 seasons), 25 were excluded because there were fewer than 3 SMIs of any type of medical institution in a prefecture. Ultimately, 116 epidemic cases were obtained.

To determine the number of SMIs needed for estimating incidence in each prefecture, we first calculated the minimum number of SMIs needed to satisfy the condition that the standard error rate of incidence estimated in the prefecture for an influenza epidemic case was less than 10% (as described below). Second, using those numbers of SMIs calculated for the 116 influenza epidemic cases, we calculated the number of SMIs required for estimating incidence in the prefecture, thereby satisfying the assumption that the standard error rate of incidence estimates for critical proportions (25%, 50%, 75%, or 90%) of influenza epidemic cases was less than 10%.

The method for calculating the minimum required number of SMIs in a prefecture to satisfy the condition that the standard error rate of an incidence estimate for an influenza epidemic case is less than 10% was as follows.^8^ The suffix of *k* from 1 to 4 indicates the first, second, third, and fourth types of medical institution, respectively. Consider an influenza epidemic case. For a type of medical institution, such as *k*, the mean and SD of the numbers of influenza patients in all medical institutions in the epidemic case are given as *μ_k_* and *σ_k_*, respectively. Let *n_k_* and *N_k_* be the number of all medical institutions and SMIs, respectively. The values of *μ_k_* and *σ_k_* were obtained: *n_k_* is known and *N_k_* is unknown. Note that the incidence is *α_k_* = *n_k_μ_k_*. The variance of an incidence estimate is given as *β_k_*^2^ = {(*N_k_* − 1)*n_k_*^3^/(*N_k_*(*n_k_* − 1))}*σ_k_*^2^(1/*N_k_* − 1/*n_k_*).^6^^,^^11^ The standard error rate of the total incidence estimate is expressed as $\sqrt{\beta_{1}{}^{2}+\beta_{2}{}^{2}+\beta_{3}{}^{2}+\beta_{4}{}^{2}}/(\alpha_{1}+\alpha_{2}+\alpha_{3}+\alpha_{4})$. We assumed that *N*_1_ and *N*_2_ were equal to the standard numbers of their SMIs (obtained by the method described in Table 1), that *N*_3_ and *N*_4_ were not less than the standard numbers of SMIs, and that the ratio of *N*_3_ to *N*_4_ was equal to the ratio of the numbers of all medical institutions. These assumptions are discussed below. Thus, from the equation that the standard error rate of the total incidence estimate was equal to 10%, *N*_3_ and *N*_4_ could be obtained by giving *N*_1_, *N*_2_ and the ratio of *N*_3_/*N*_4_. The total number of SMIs in all types of medical institution was estimated as *N*_1_ + *N*_2_ + *N*_3_ + *N*_4_.

## RESULTS

Table 2 shows the distributions of the numbers of influenza patients in SMIs among the 116 epidemic cases. In hospital pediatrics departments, the mean, SD, and coefficient of variation of the numbers of influenza patients in SMIs ranged widely among the epidemic cases. Median, 25th, and 75th percentiles of the mean numbers of influenza patients in SMIs among the 116 epidemic cases were 262, 159, and 402, respectively. Median, 25th, and 75th percentiles of the SDs were 192, 127, and 319. Median, 25th, and 75th percentiles of the coefficients of variation were 79%, 66%, and 91%. In other types of medical institutions, the mean, SD, and coefficient of variation of the numbers of influenza patients in SMIs ranged widely among epidemic cases.

**Table 2.** Distributions of numbers of influenza patients in SMIs among 116 epidemic cases

Number of influenza patients in SMIs	Type of medical institution^a^			

	Type 1	Type 2	Type 3	Type 4
Means				
Minimum^b^	66.0	97.7	49.6	26.8
25th percentile	159.1	242.3	112.6	96.2
Median	262.0	370.8	197.5	147.9
75th percentile	402.3	503.7	275.1	204.2
Maximum	1553.1	927.3	426.2	794.1

Standard deviations				
Minimum^b^	30.5	69.0	22.7	24.5
25th percentile	126.7	144.8	83.4	81.0
Median	192.0	234.3	159.3	141.8
75th percentile	319.3	314.0	238.0	213.7
Maximum	1631.7	887.2	667.8	719.2

Coefficients of variation (%)				
Minimum^b^	23.3	29.8	21.1	57.1
25th percentile	66.4	55.2	66.6	78.8
Median	78.5	65.3	87.9	94.8
75th percentile	90.8	73.2	105.5	113.8
Maximum	191.2	133.1	184.9	223.5

SMI: sentinel medical institution.

^a^Institution types are defined in the Methods.

^b^Minimum, maximum, median, 25th, and 75th percentiles for the 116 epidemic cases.

As mentioned above, we assumed that the numbers of SMIs needed for estimating influenza incidence in prefectures were equal to their standard numbers of SMIs in hospital pediatrics departments and pediatrics clinics. Table 3 shows the numbers of all medical institutions and the standard numbers of SMIs in prefectures. In hospital pediatrics departments, the standard numbers of SMIs in prefectures ranged from 5 to 65, and the total was 968 (38.7% of all medical institutions). In pediatrics clinics, the standard numbers of SMIs in prefectures ranged from 11 to 221, and the total was 2140 (38.2%).

**Table 3.** Numbers of all medical institutions and standard numbers of SMIs in hospital pediatrics departments and pediatrics clinics

Prefecture	Hospital pediatrics departments		Pediatrics clinics	
	
	No. of all medical institutions	Standard no. of SMIs	No. of all medical institutions	Standard no. of SMIs
Hokkaido	146	58 (39.7)	213	85 (39.9)
Aomori	33	14 (42.4)	50	21 (42.0)
Iwate	41	17 (41.5)	50	21 (42.0)
Miyagi	42	18 (42.9)	96	42 (43.8)
Akita	26	12 (46.2)	45	21 (46.7)
Yamagata	24	9 (37.5)	57	20 (35.1)
Fukushima	43	16 (37.2)	95	34 (35.8)
Ibaraki	76	35 (46.1)	83	38 (45.8)
Tochigi	34	15 (44.1)	73	32 (43.8)
Gunma	36	12 (33.3)	114	40 (35.1)

Saitama	110	45 (40.9)	271	111 (41.0)
Chiba	96	41 (42.7)	226	95 (42.0)
Tokyo	175	59 (33.7)	649	221 (34.1)
Kanagawa	106	42 (39.6)	420	168 (40.0)
Niigata	55	21 (38.2)	102	40 (39.2)
Toyama	33	10 (30.3)	57	17 (29.8)
Ishikawa	38	12 (31.6)	56	17 (30.4)
Fukui	29	10 (34.5)	37	12 (32.4)
Yamanashi	23	10 (43.5)	29	13 (44.8)
Nagano	63	26 (41.3)	75	30 (40.0)

Gifu	47	16 (34.0)	102	34 (33.3)
Shizuoka	51	21 (41.2)	160	64 (40.0)
Aichi	116	44 (37.9)	342	130 (38.0)
Mie	40	17 (42.5)	72	30 (41.7)
Shiga	31	12 (38.7)	60	22 (36.7)
Kyoto	64	25 (39.1)	118	47 (39.8)
Osaka	138	65 (47.1)	326	153 (46.9)
Hyogo	92	32 (34.8)	286	99 (34.6)
Nara	27	11 (40.7)	56	23 (41.1)
Wakayama	25	9 (36.0)	58	20 (34.5)

Tottori	16	5 (31.3)	35	11 (31.4)
Shimane	25	8 (32.0)	38	12 (31.6)
Okayama	51	19 (37.3)	71	27 (38.0)
Hiroshima	58	22 (37.9)	135	50 (37.0)
Yamaguchi	37	13 (35.1)	71	26 (36.6)
Tokushima	35	11 (31.4)	37	11 (29.7)
Kagawa	28	11 (39.3)	38	15 (39.5)
Ehime	28	10 (35.7)	78	27 (34.6)
Kouchi	32	12 (37.5)	29	10 (34.5)
Fukuoka	83	29 (34.9)	265	91 (34.3)

Saga	25	8 (32.0)	42	14 (33.3)
Nagasaki	39	13 (33.3)	84	27 (32.1)
Kumamoto	50	18 (36.0)	82	29 (35.4)
Oita	33	12 (36.4)	50	18 (36.0)
Miyazaki	26	11 (42.3)	49	21 (42.9)
Kagoshima	40	20 (50.0)	61	30 (49.2)
Okinawa	36	12 (33.3)	65	21 (32.3)

Totals	2502	968 (38.7)	5608	2140 (38.2)

SMI: sentinel medical institution.

Percentages of all medical institutions are in parentheses.

We calculated the numbers of SMIs needed for estimating influenza incidence in prefectures, assuming that the standard error rate of incidence estimates for critical proportions (25%, 50%, 75%, or 90%) of influenza epidemic cases was less than 10%. The numbers of SMIs are described as “the numbers of SMIs calculated for critical proportions (25%, 50%, 75%, or 90%) of epidemic cases”. Table 4 shows the numbers of all medical institutions, the standard numbers of SMIs, and the numbers of SMIs needed for estimating prefectural influenza incidence in internal medicine clinics with a secondary pediatrics practice. The standard numbers of prefectural SMIs ranged from 3 to 41, and the total was 490 (3.3% of all medical institutions). The numbers of prefectural SMIs for a critical proportion of 25% of epidemic cases ranged from 5 to 43, and the values in 32 prefectures were greater than the standard numbers of SMIs. The numbers of prefectural SMIs for a critical proportion of 75% of epidemic cases ranged from 9 to 43 and were equal to the standard numbers of SMIs in 9 prefectures, namely, Hokkaido, Saitama, Chiba, Tokyo, Kanagawa, Aichi, Osaka, Hyogo, and Fukuoka. The total numbers of SMIs needed for critical proportions of 25%, 50%, 75%, and 90% of epidemic cases were 629 (4.3%), 770 (5.2%), 910 (6.2%), and 1155 (7.8%), respectively.

**Table 4.** Numbers of all medical institutions, standard numbers of SMIs, and numbers of SMIs needed for estimating prefectural influenza incidence: internal medicine clinics with a secondary pediatric practice

Prefecture	No. of all medical institutions	Standard no. of SMIs	No. of SMIs needed for estimating influenza incidence in prefectures^a^			

			Critical proportion of influenza epidemic cases			
			25%	50%	75%	90%
Hokkaido	428	17 (4.0)	17 (4.0)	17 (4.0)	17 (4.0)	17 (4.0)
Aomori	183	7 (3.8)	12 (6.6)	17 (9.3)	21 (11.5)	29 (15.8)
Iwate	81	3 (3.7)	5 (6.2)	7 (8.6)	9 (11.1)	13 (16.0)
Miyagi	229	9 (3.9)	9 (3.9)	13 (5.7)	16 (7.0)	22 (9.6)
Akita	94	4 (4.3)	7 (7.4)	9 (9.6)	12 (12.8)	16 (17.0)
Yamagata	106	3 (2.8)	7 (6.6)	10 (9.4)	13 (12.3)	17 (16.0)
Fukushima	297	9 (3.0)	11 (3.7)	17 (5.7)	20 (6.7)	29 (9.8)
Ibaraki	348	13 (3.7)	13 (3.7)	16 (4.6)	21 (6.0)	30 (8.6)
Tochigi	269	9 (3.3)	13 (4.8)	18 (6.7)	22 (8.2)	31 (11.5)
Gunma	295	9 (3.1)	11 (3.7)	16 (5.4)	19 (6.4)	27 (9.2)

Saitama	746	29 (3.9)	29 (3.9)	29 (3.9)	29 (3.9)	29 (3.9)
Chiba	607	24 (4.0)	24 (4.0)	24 (4.0)	24 (4.0)	27 (4.4)
Tokyo	1718	41 (2.4)	41 (2.4)	41 (2.4)	41 (2.4)	41 (2.4)
Kanagawa	731	31 (4.2)	31 (4.2)	31 (4.2)	31 (4.2)	31 (4.2)
Niigata	227	8 (3.5)	8 (3.5)	12 (5.3)	15 (6.6)	20 (8.8)
Toyama	105	3 (2.9)	7 (6.7)	10 (9.5)	13 (12.4)	17 (16.2)
Ishikawa	88	3 (3.4)	6 (6.8)	8 (9.1)	11 (12.5)	14 (15.9)
Fukui	124	4 (3.2)	12 (9.7)	17 (13.7)	21 (16.9)	29 (23.4)
Yamanashi	137	5 (3.6)	14 (10.2)	19 (13.9)	23 (16.8)	31 (22.6)
Nagano	323	11 (3.4)	13 (4.0)	18 (5.6)	22 (6.8)	32 (9.9)

Gifu	436	14 (3.2)	18 (4.1)	24 (5.5)	32 (7.3)	40 (9.2)
Shizuoka	323	11 (3.4)	11 (3.4)	12 (3.7)	16 (5.0)	21 (6.5)
Aichi	1110	43 (3.9)	43 (3.9)	43 (3.9)	43 (3.9)	43 (3.9)
Mie	242	7 (2.9)	10 (4.1)	15 (6.2)	19 (7.9)	26 (10.7)
Shiga	206	7 (3.4)	13 (6.3)	17 (8.3)	22 (10.7)	30 (14.6)
Kyoto	356	12 (3.4)	12 (3.4)	14 (3.9)	19 (5.3)	25 (7.0)
Osaka	970	31 (3.2)	31 (3.2)	31 (3.2)	31 (3.2)	31 (3.2)
Hyogo	571	18 (3.2)	18 (3.2)	18 (3.2)	18 (3.2)	21 (3.7)
Nara	186	6 (3.2)	12 (6.5)	17 (9.1)	20 (10.8)	28 (15.1)
Wakayama	163	4 (2.5)	9 (5.5)	13 (8.0)	17 (10.4)	22 (13.5)

Tottori	102	3 (2.9)	11 (10.8)	16 (15.7)	20 (19.6)	26 (25.5)
Shimane	155	4 (2.6)	13 (8.4)	18 (11.6)	22 (14.2)	30 (19.4)
Okayama	353	9 (2.5)	14 (4.0)	20 (5.7)	25 (7.1)	35 (9.9)
Hiroshima	296	9 (3.0)	9 (3.0)	11 (3.7)	14 (4.7)	19 (6.4)
Yamaguchi	136	4 (2.9)	7 (5.1)	9 (6.6)	12 (8.8)	16 (11.8)
Tokushima	184	4 (2.2)	14 (7.6)	19 (10.3)	23 (12.5)	32 (17.4)
Kagawa	88	3 (3.4)	7 (8.0)	10 (11.4)	13 (14.8)	17 (19.3)
Ehime	110	4 (3.6)	6 (5.5)	8 (7.3)	10 (9.1)	14 (12.7)
Kouchi	68	3 (4.4)	6 (8.8)	8 (11.8)	11 (16.2)	14 (20.6)
Fukuoka	477	15 (3.1)	15 (3.1)	15 (3.1)	15 (3.1)	19 (4.0)

Saga	124	4 (3.2)	10 (8.1)	14 (11.3)	17 (13.7)	24 (19.4)
Nagasaki	166	5 (3.0)	8 (4.8)	11 (6.6)	14 (8.4)	19 (11.4)
Kumamoto	241	8 (3.3)	9 (3.7)	14 (5.8)	18 (7.5)	23 (9.5)
Oita	124	4 (3.2)	8 (6.5)	10 (8.1)	14 (11.3)	18 (14.5)
Miyazaki	92	3 (3.3)	6 (6.5)	8 (8.7)	11 (12.0)	14 (15.2)
Kagoshima	215	7 (3.3)	9 (4.2)	13 (6.0)	17 (7.9)	23 (10.7)
Okinawa	140	6 (4.3)	10 (7.1)	13 (9.3)	17 (12.1)	23 (16.4)

Totals	14 770	490 (3.3)	629 (4.3)	770 (5.2)	910 (6.2)	1155 (7.8)

SMI: sentinel medical institution.

Percentages of all medical institutions are in parentheses.

^a^Numbers of SMIs for estimating influenza incidences in prefectures were calculated with the assumption that the standard error rate of the incidence estimate for a given critical proportion (25%, 50%, 75%, or 90%) of influenza epidemic cases was less than 10%.

Table 5 shows the numbers of all medical institutions, the standard numbers of SMIs, and the numbers of SMIs needed for estimating prefectural influenza incidence in hospital departments of internal medicine and internal medicine clinics with no pediatrics practice. The standard numbers of SMIs in prefectures ranged from 8 to 120, and the total was 1403 (3.3% of all medical institutions). The numbers of SMIs needed for a critical proportion of 25% of epidemic cases in prefectures ranged from 21 to 120, and the values in 32 prefectures were greater than the standard numbers of SMIs. The numbers of SMIs needed for a critical proportion of 75% of epidemic cases in prefectures ranged from 38 to 120 and were equal to the standard numbers of SMIs in 9 prefectures. The total numbers of SMIs needed for critical proportions of 25%, 50%, 75%, and 90% of epidemic cases were 1811 (4.2%), 2234 (5.2%), 2651 (6.2%), and 3387 (7.9%), respectively.

**Table 5.** Numbers of all medical institutions, standard numbers of SMIs, and numbers of SMIs needed for estimating influenza incidence in prefectures: hospital departments of internal medicine and internal medicine clinics with no pediatric practice

Prefecture	No. of all medical institutions	Standard no. of SMIs	No. of SMIs needed for estimating influenza incidence in prefectures^a^			

			Critical proportion of influenza epidemic cases			
			25%	50%	75%	90%
Hokkaido	1652	68 (4.1)	68 (4.1)	68 (4.1)	68 (4.1)	68 (4.1)
Aomori	380	15 (3.9)	26 (6.8)	36 (9.5)	44 (11.6)	61 (16.1)
Iwate	485	21 (4.3)	31 (6.4)	44 (9.1)	55 (11.3)	75 (15.5)
Miyagi	705	28 (4.0)	28 (4.0)	40 (5.7)	50 (7.1)	68 (9.6)
Akita	423	18 (4.3)	30 (7.1)	43 (10.2)	56 (13.2)	72 (17.0)
Yamagata	472	14 (3.0)	31 (6.6)	43 (9.1)	56 (11.9)	75 (15.9)
Fukushima	711	21 (3.0)	27 (3.8)	40 (5.6)	49 (6.9)	68 (9.6)
Ibaraki	817	31 (3.8)	31 (3.8)	38 (4.7)	48 (5.9)	71 (8.7)
Tochigi	604	19 (3.1)	28 (4.6)	40 (6.6)	49 (8.1)	69 (11.4)
Gunma	699	23 (3.3)	26 (3.7)	38 (5.4)	46 (6.6)	64 (9.2)

Saitama	1577	60 (3.8)	60 (3.8)	60 (3.8)	60 (3.8)	62 (3.9)
Chiba	1436	56 (3.9)	56 (3.9)	56 (3.9)	56 (3.9)	64 (4.5)
Tokyo	5074	120 (2.4)	120 (2.4)	120 (2.4)	120 (2.4)	120 (2.4)
Kanagawa	2310	99 (4.3)	99 (4.3)	99 (4.3)	99 (4.3)	99 (4.3)
Niigata	803	28 (3.5)	28 (3.5)	41 (5.1)	52 (6.5)	69 (8.6)
Toyama	418	14 (3.3)	29 (6.9)	40 (9.6)	52 (12.4)	70 (16.7)
Ishikawa	455	16 (3.5)	29 (6.4)	42 (9.2)	55 (12.1)	74 (16.3)
Fukui	242	9 (3.7)	24 (9.9)	32 (13.2)	40 (16.5)	57 (23.6)
Yamanashi	260	10 (3.8)	26 (10.0)	35 (13.5)	44 (16.9)	59 (22.7)
Nagano	654	22 (3.4)	26 (4.0)	36 (5.5)	44 (6.7)	66 (10.1)

Gifu	519	17 (3.3)	21 (4.0)	28 (5.4)	38 (7.3)	47 (9.1)
Shizuoka	1107	37 (3.3)	37 (3.3)	42 (3.8)	53 (4.8)	71 (6.4)
Aichi	1698	66 (3.9)	66 (3.9)	66 (3.9)	66 (3.9)	66 (3.9)
Mie	694	21 (3.0)	30 (4.3)	43 (6.2)	55 (7.9)	75 (10.8)
Shiga	422	15 (3.6)	26 (6.2)	36 (8.5)	45 (10.7)	62 (14.7)
Kyoto	1053	36 (3.4)	36 (3.4)	42 (4.0)	55 (5.2)	74 (7.0)
Osaka	3227	103 (3.2)	103 (3.2)	103 (3.2)	103 (3.2)	103 (3.2)
Hyogo	1927	59 (3.1)	59 (3.1)	59 (3.1)	59 (3.1)	72 (3.7)
Nara	442	14 (3.2)	28 (6.3)	40 (9.0)	48 (10.9)	66 (14.9)
Wakayama	575	14 (2.4)	32 (5.6)	46 (8.0)	60 (10.4)	78 (13.6)

Tottori	242	8 (3.3)	27 (11.2)	37 (15.3)	46 (19.0)	61 (25.2)
Shimane	347	9 (2.6)	29 (8.4)	40 (11.5)	49 (14.1)	67 (19.3)
Okayama	711	18 (2.5)	29 (4.1)	40 (5.6)	50 (7.0)	71 (10.0)
Hiroshima	1303	38 (2.9)	38 (2.9)	47 (3.6)	63 (4.8)	82 (6.3)
Yamaguchi	668	20 (3.0)	32 (4.8)	45 (6.7)	59 (8.8)	79 (11.8)
Tokushima	396	9 (2.3)	29 (7.3)	40 (10.1)	49 (12.4)	68 (17.2)
Kagawa	378	13 (3.4)	32 (8.5)	43 (11.4)	55 (14.6)	73 (19.3)
Ehime	624	20 (3.2)	31 (5.0)	45 (7.2)	58 (9.3)	80 (12.8)
Kouchi	387	11 (2.8)	34 (8.8)	47 (12.1)	60 (15.5)	80 (20.7)
Fukuoka	1854	58 (3.1)	58 (3.1)	58 (3.1)	58 (3.1)	72 (3.9)

Saga	365	11 (3.0)	30 (8.2)	41 (11.2)	51 (14.0)	70 (19.2)
Nagasaki	629	20 (3.2)	29 (4.6)	41 (6.5)	53 (8.4)	72 (11.4)
Kumamoto	746	23 (3.1)	29 (3.9)	42 (5.6)	54 (7.2)	73 (9.8)
Oita	541	15 (2.8)	34 (6.3)	45 (8.3)	59 (10.9)	79 (14.6)
Miyazaki	525	18 (3.4)	34 (6.5)	48 (9.1)	62 (11.8)	81 (15.4)
Kagoshima	779	24 (3.1)	33 (4.2)	48 (6.2)	61 (7.8)	82 (10.5)
Okinawa	318	14 (4.4)	22 (6.9)	31 (9.7)	39 (12.3)	52 (16.4)

Totals	42 654	1403 (3.3)	1811 (4.2)	2234 (5.2)	2651 (6.2)	3387 (7.9)

SMI: sentinel medical institution.

Percentages of all medical institutions are in parentheses.

^a^Numbers of SMIs for estimating influenza incidences in prefectures were calculated with the assumption that the standard error rate of the incidence estimate for a given critical proportion (25%, 50%, 75%, or 90%) of influenza epidemic cases was less than 10%.

The total number of SMIs among all types of medical institutions was 5001. The total numbers of SMIs needed for critical proportions of 25%, 50%, 75%, and 90% of epidemic cases, for all types of medical institutions, were 5548, 6112, 6669, and 7650, respectively. The Figure shows the standard numbers of SMIs and the numbers of SMIs needed for estimating prefectural influenza incidence, for all types of medical institutions. The differences between the standard numbers of SMIs and the proposed numbers of SMIs for a critical proportion of 75% of epidemic cases in prefectures ranged from 0 to 59, and the total was 1668.

**Figure.**
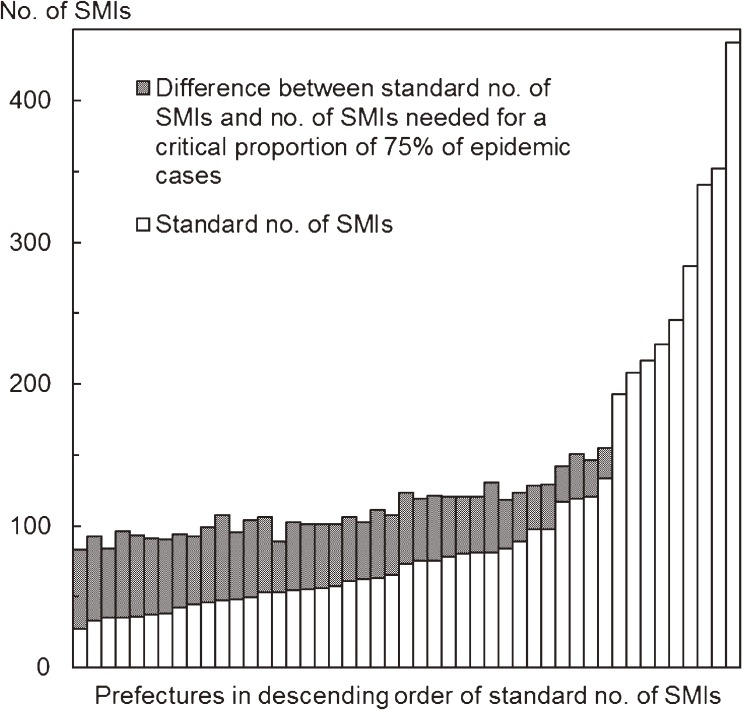
The standard numbers of SMIs and the numbers of SMIs needed for estimating prefectural influenza incidence, for all types of medical institutions. SMI: sentinel medical institution.

## DISCUSSION

### Influenza surveillance and numbers of SMIs

The Japanese NESID guidelines specify the method to be used for determining the standard number of SMIs in the influenza surveillance system.^7^^,^^9^ The aim of the guidelines is to facilitate detection of epidemics in local areas and incidence estimation nationwide rather than by prefecture.^6^^,^^16^ In reality, the numbers of SMIs selected in prefectures were nearly equal to those specified in the standards.^17^ Nationwide annual incidence estimates of influenza have been reported, and their standard error rates were found to be less than 5%.^6^^,^^11^^,^^12^

### Numbers of SMIs and precision of prefectural influenza incidence estimates

In 32 of the 47 prefectures, the standard number of SMIs in the prefecture was less than the number of SMIs needed for a critical proportion of 25% of epidemic cases, which means that the standard error rates for influenza incidence estimates in those prefectures would frequently (>75% of epidemic cases) exceed 10% and suggests that, under the current surveillance system, the precision of influenza incidence estimates would be insufficient in many prefectures. We calculated the numbers of SMIs needed for estimating prefectural influenza incidence under the assumption that the standard error rate of the incidence estimate in 75% of influenza epidemic cases was less than 10%. We propose that prefectural governments increase the number of SMIs to the levels we have specified, as the standard error rates of influenza incidence estimates would then frequently (>75% of epidemic cases) be less than 10%.

### Increase of SMIs in prefectures and feasibility

Our proposal would increase the number of internal medicine clinics with a secondary pediatrics practice (third type of medical institution) and hospital departments of internal medicine and internal medicine clinics with no pediatrics practice (fourth type of medical institution). We assumed that the numbers of SMIs needed for estimating influenza incidence in prefectures were equal to the standard numbers of SMIs in the other 2 types of medical institutions, namely, hospital pediatrics departments and pediatric clinics. The increase from the standard numbers of SMIs in prefectures ranged from 0 to 59 for the third and fourth types of medical institutions. For these 2 types of medical institutions, the proportion of SMIs to all medical institutions in prefectures that implemented our proposal would be less than 17% (as shown in Tables 4 and 5), which is less than the proportion of the first and second types of medical institution (≥30% of the standard numbers of SMIs shown in Table 3).^8^^,^^15^ Thus, an increase in prefectural SMIs in accordance with our proposal is feasible.

### Main assumption for determining the number of SMIs needed for estimating prefectural influenza incidence

The standard error rate is commonly used as an index of estimate precision.^8^^,^^18^ A primary assumption of the present study is that the standard error rate of the incidence estimate in 75% of influenza epidemic cases was less than 10%, as that seemed an appropriate level of precision for producing prefectural influenza incidence estimates.^19^ For example, when total incidence is 100 000 and the standard error rate of the estimate is 10%, the approximate 95% confidence interval is 80 000 to 120 000.^6^ In a previous study of the numbers of SMIs needed to estimate nationwide incidences in Japan, the standard error rate was 5% for sentinel surveillances of influenza and pediatric diseases and 10% for sentinel surveillances of ophthalmologic and sexually transmitted diseases.^8^ A standard error rate of 5% has frequently been used for determining the sample size of surveys in health statistics.^18^ A previous study used a standard error rate of 10% in consideration of the minimum required level of precision for incidence estimates and the feasibility of selecting SMIs in prefectures.^8^ In the present study, the total number of SMIs required for estimating prefectural incidence, assuming a standard error rate of 10%, was 6669, and the total increase from the standard number of SMIs was 1668. In contrast, if we used a 5% standard error rate for influenza incidence estimates in each prefecture, the total number of SMIs required would be greater than 10 000, which is not feasible.

Under the primary assumption described above, the critical proportion of influenza epidemic cases was 75%. We considered 75% of epidemic cases as the proportion needed to maintain the precision of incidence estimates above a desirable threshold. There were no definite reasons for using a critical proportion of 75%. The numbers of SMIs needed for 50% and 90% of influenza epidemic cases are therefore presented in Tables 4 and 5, respectively. If the critical proportion of epidemic cases was higher or lower than 75% (eg, 50% or 90%), the numbers of SMIs needed increased or decreased. The influenza epidemic cases observed in 47 prefectures in 3 seasons were used and are more appropriate than hypothetical cases.^8^ Although the epidemic cases included epidemics of A(H1N1)pdm09,^14^ the median, 25th, and 75th percentiles of means and SDs of the numbers of influenza patients in SMIs among epidemic cases, not including the 2009/2010 season, did not change greatly.

### Other assumptions in determining the number of SMIs needed for estimating prefectural influenza incidence

We made another assumption, ie, that the numbers of SMIs needed for estimating prefectural influenza incidences were equal to the standard numbers of SMIs in hospital pediatrics departments and pediatrics clinics. One reason for this assumption is that the standard numbers of SMIs were sufficiently large. Without this assumption, the numbers of SMIs in these 2 types of medical institutions would be at or below the standard numbers of SMIs for almost all prefectures. Another reason was that proposing SMI numbers that were less than the standards would not be reasonable. The standards were determined by considering the several roles of SMIs in these 2 types of medical institutions, such as detection of epidemics of pediatric infectious diseases in local areas.^6^^,^^7^^,^^20^

We divided the standard numbers of pediatrics SMIs into 2 types of medical institutions (hospital pediatrics departments and pediatrics clinics), proportional to the numbers of all medical institutions. We did this to adhere to NESID guidelines, which specify that SMIs should be selected as randomly and as representatively as possible from among all medical institutions in an area.^7^^,^^9^ For the same reason, we assumed that the ratio of SMIs in the other 2 types of medical institutions (internal medicine clinics with a secondary pediatrics practice and hospital internal medicine departments/internal medicine clinics with no pediatrics practice) was equal to the ratio among all medical institutions.

### Problems and limitations

We attempted to obtain accurate estimates of influenza incidence in each prefecture, using influenza sentinel surveillance data from Japan. A critical problem in achieving this goal is accurately diagnosing influenza. Surveillance guidelines specify the case definition of influenza.^7^^,^^8^ When estimating influenza incidence, a key assumption is that SMIs are randomly selected from all medical institutions.^6^^,^^11^ However, SMI recruitment is to some extent voluntary.^6^^,^^17^ We believe that it is not sufficient to evaluate bias in incidence estimates caused by violation of this assumption.^6^^,^^11^ Surveys of all influenza patients at all medical institutions in selected areas would provide useful information. Additional detailed studies are therefore warranted.

### Conclusion

We calculated the numbers of SMIs needed to estimate prefectural influenza incidences in the NESID in Japan, assuming a standard error rate of less than 10% for 75% of influenza epidemic cases. The total number of SMIs needed was 6669. The increase from the standard number of SMIs required by NESID guidelines ranged from 0 to 59 in prefectures, and the total number needed was 1668. The standard error rate of an estimate of influenza incidence would frequently (>75% of epidemic cases) be less than 10% in prefectures with the proposed number of SMIs but would frequently be greater than 10% in many prefectures that have only the standard number of SMIs.

## ONLINE ONLY MATERIALS

Abstract in Japanese.

## APPENDIX

### Method for estimating incidence

The method for estimating incidence is as follows.^6^^,^^11^ Consider the distribution of incidences in medical institutions. Let *m* be an integer greater than the largest incidence among medical institutions, *n* be the number of all medical institutions, and *n_i_* be the number of medical institutions with an incidence of *i* for *i* = 0, 1, …, *m*. Let *N* be the number of SMIs and *N_i_* be the number of SMIs with an incidence of *i* for *i* = 0, 1, …, *m*. The constants of *n* and *N* are known, and those of {*n_i_*} are unknown. {*N_i_*} are obtained from sentinel surveillance and follow a multi-hypergeometric distribution under the condition that *N* is fixed under the assumption that SMIs are randomly selected in all medical institutions.

Let *α* be the total incidence in all medical institutions, and note that *α* = Σ*i***n_i_*. The estimate of *α* is expressed as *α*^ = Σ*i***N_i_***n*/*N*, ie, the incidence is estimated as the total incidence in SMIs (Σ*i***N_i_*) divided by the proportion of SMIs among all medical institutions (*N*/*n*).

Consider that incidences in some strata, such as type of medical institution, are estimated using the above method. Let *k* be the number of strata and *α*_1_^, *α*_2_^, …, *α_k_*^ the estimated incidences in the strata. The total incidence is estimated as *α_t_*^ = *α*_1_^ + *α*_2_^ + … + *α_k_*^.
