# Supplementary material for: Number of Sentinel Medical Institutions Needed for Estimating Prefectural Incidence in Influenza Surveillance in Japan
Source: J Epidemiol. 2014 May 5;24(3):183–92. doi: 10.2188/jea.JE20130077 (PMC4000765; doi:10.2188/jea.JE20130077)
Supplement: Abstract in Japanese. [file je-24-183-s001.pdf]

日本のインフルエンザ・サーベランスにおける都道府県罹患数推計に必要な定点医療施設数

橋本修二[1]、川戸美由紀[1]、村上義孝[2]、太田晶子[3]、重松美加[4]、多田有希[4]、谷口清州[5]、永井正規[3]

[1]藤田保健衛生大学医学部衛生学講座、[2]滋賀医科大学医学部医療統計学部門、[3]埼玉医科大学医学部公衆衛生学講座、[4]国立感染症研究所感染症疫学センター、[5]国立病院機構三重病院臨床研究部

【背景】インフルエンザの全国罹患数推計値は感染症発生動向調査システムから得られる。現行システムのデータから都道府県別罹患数が推計できるが、その精度は十分でない。

【方法】感染症発生動向調査における都道府県別インフルエンザ罹患数推計に必要な定点医療施設数を算定した。その算定にあたって、インフルエンザ流行ケースの 75％において、インフルエンザ罹患数推計値の標準誤差率が 10％未満となることを仮定した。インフルエンザ流行ケースとして、3 シーズン（2007/2008 年、2008/2009 年、2009/2010 年）の 47 都道府県で観察されたものを用いた。

【結果】定点医療施設の標準数は 6,669 であった。都道府県別インフルエンザ罹患数推計に必要な定点医療施設数として、この標準数からの増加は各都道府県で 0～59 施設、合計で 1,668 施設であった。

【結論】感染症発生動向調査データに基づいて、各都道府県におけるインフルエンザ罹患数推計に必要な定点医療施設数を求めた。

キーワード：サーベイランス、感染症、インフルエンザ、疫学
